# Evaluation of antibacterial and cytotoxic effects of silver oxide nanoparticles synthesized from *Psidium Guajava*

**DOI:** 10.1038/s41598-025-24924-6

**Published:** 2025-11-25

**Authors:** Abdullah Yousef, Salem S. Salem, Mohammad M. Torayah, Sara A. Saied, Rania Hamed Elbawab

**Affiliations:** 1 Basic & Medical Sciences Department, Faculty of Dentistry, Al-Ryada University for Science and Technology, Sadat City, Egypt; 2 Department of Botany and Microbiology, Faculty of Science, Al-Azhar University, Nasr City, Cairo 11884 Egypt; 3 Department of Anaesthesia and Intensive Care, Critical Care Unit, Faculty of Medicine, Menoufia University Shebin El‑Kom, Shebeen El-Kom, Egypt; 4 Department of Clinical Pathology, National Liver Institute, Menoufia University, Shebin El-Kom, Egypt; 5 Medical Laboratory Department, Medical Administration, University of Sadat City, Sadat City, Egypt

**Keywords:** Silver oxide nanoparticles, Multi-drug-resistant bacteria- antimicrobial activity, Anticancer activity – cytotoxicity on normal cells, Biochemistry, Biotechnology, Cancer, Chemistry, Drug discovery, Microbiology, Nanoscience and technology

## Abstract

Growing concerns over the toxicity and environmental impact of traditional nanoparticle synthesis methods have driven the search for safer, more sustainable alternatives. At the same time, the rising prevalence of antibiotic-resistant bacteria and the ongoing challenges in effective cancer treatment emphasize the urgent need for new antimicrobial and anticancer solutions. The manufacture of silver oxide nanoparticles (Ag₂O-NPs) is investigated in this work using *Psidium guajava* (guava) leaves extract, focusing on their dual biological potential. The use of dangerous chemicals is reduced by this green synthesis technique by employing natural phytochemicals from guava leaves as stabilizing and reducing agents, making it more environmentally friendly compared to traditional chemical approaches. At 435 nm, the biosynthesized Ag₂O-NPs displayed a distinctive surface plasmon resonance (SPR) band. With a polydispersity index (PDI) of 0.368. Nanocrystalline, mostly spherical Ag₂O-NPs with an average size of 25 to 30 nm were successfully formed, according to thorough characterization utilizing FTIR, XRD, SEM, TEM, and EDX. These nanoparticles demonstrated strong antibacterial activity against four clinically relevant, drug-resistant bacterial strains: *Pseudomonas aeruginosa* ATCC 27853, *Salmonella typhimurium* ATCC 13311, *Escherichia coli* ATCC 25922, *and Staphylococcus aureus* ATCC 29213. Minimum inhibitory concentration (MIC) values ranged from 31.2 to 250 µg/mL, indicating a dose-dependent antibacterial action. Notably, *P. aeruginosa* and *S. aureus* showed the highest sensitivity. Cytotoxicity testing further revealed selective anticancer activity, with the Ag₂O-NPs significantly reducing the viability of HepG2 liver cancer cells (IC₅₀ = 73.93 ± 0.49 µg/mL), while displaying lower toxicity toward normal Vero cells (IC₅₀ = 158.1 ± 0.41 µg/mL). These findings suggest that green-synthesized Ag₂O-NPs hold considerable promise as both potent antibacterial agents and effective anticancer therapeutics.

**Supplementary Information:**

The online version contains supplementary material available at 10.1038/s41598-025-24924-6.

## Introduction

Antibiotic resistance and cancer remain two of the most pressing global health challenges, demanding the exploration of new therapeutic strategies. Conventional methods for nanoparticle synthesis often rely on toxic chemicals and harsh conditions that raise environmental and safety concerns^1–4^. In response, green synthesis has emerged as a sustainable approach, utilizing plant-derived phytochemicals as reducing and stabilizing agents^5–8^. This eco-friendly method not only minimizes environmental hazards but also produces nanoparticles with enhanced biocompatibility and potential biomedical applications^9–12^.

Silver oxide nanoparticles (Ag₂O-NPs) are one type of nanomaterial that has garnered a lot of interest because of its potential anticancer and broad-spectrum antibacterial qualities. According to numerous research, Ag₂O-NPs made from plant extracts have strong antibacterial action by rupturing cell membranes, producing reactive oxygen species, and obstructing DNA replication^13,14^. In addition, Ag₂O-NPs have been demonstrated to exert selective cytotoxic effects against cancer cells, including HepG2 liver carcinoma, while demonstrating reduced toxicity to normal cells^15^. These results demonstrate Ag₂O-NPs’ dual therapeutic potential in treating cancer and infectious illnesses.

Phytochemicals like flavonoids, tannins, and phenolic compounds found in *Psidium guajava* (guava) leaves can serve as organic capping and reducing agents. Guava extracts have been shown in earlier studies to be used in the environmentally friendly manufacture of metallic nanoparticles with strong antibacterial properties^16,17^. The potential of plant-derived biowastes for sustainable nanomaterial synthesis is further supported by recent research. For example, orange fruit peel can be utilized to generate environmentally friendly CaO nanoparticles with antibacterial, radical-scavenging, and eco-toxicological properties^18^. Similarly, ZnO nanoparticles synthesized from *Lawsonia inermis* demonstrated enhanced bactericidal, antibiofilm, and antioxidative activities when combined with commercial antibiotics^19^. *Tamarindus indica* fruit extract-derived iron oxide nanoparticles efficiently scavenged free radicals and inhibited microorganisms that produce biofilms^20^, while silver nanoparticles synthesized from *Mentha spicata* leaves exhibited antibacterial, antibiofilm, and cytotoxicity activities^21^. Further examples include multifunctional nanoparticles produced using *Carica papaya* fruit extract^22^, *Aegle marmelos* unripe fruit extract^23^, and *Withania somnifera* leaves^24^, all of which showed strong antibacterial, antioxidant, and antibiofilm responses. In addition, Ag-NPs derived from *Solanum nigrum*^25^ and ZnO NPs from *Pongamia pinnata*^26^ highlighted the therapeutic and environmental relevance of phytochemical-assisted nanomaterials.

The application of *P. guajava* in the manufacture of Ag₂O-NPs and their combination antibacterial and anticancer properties, however, have not been well investigated. Thus, the goal of the current study was to create Ag₂O-NPs using guava leaf extract, describe their physicochemical characteristics, and assess their cytotoxicity on HepG2 liver cancer cells and normal Vero cells in addition to their antibacterial effectiveness against drug-resistant infections. As far as we are aware, this work is one of the first to describe the environmentally friendly production of Ag₂O-NPs using guava leaf extract and to thoroughly assess their dual bioactivity against HepG2 liver cancer cells and pathogenic reference strains. This novelty highlights the potential of *P. guajava*-mediated Ag₂O-NPs as multifunctional therapeutic agents.

## Materials and methods

### Chemicals and silver oxide nanoparticle materials

Silver nitrate (AgNO₃), with a purity of at least 99.5%, was purchased from Sigma-Aldrich in Egypt. Fresh guava leaves were collected from the Ministry of Agriculture in Giza. Their plant identity was confirmed by the Botany Department at the Faculty of Science, Al-Azhar University, based on their physical traits.

### Preparation of guava leaf extract

Fresh *Psidium guajava* leaves were gathered from the Ministry of Agriculture in Giza, Egypt, and their botanical identity was confirmed by the Department of Botany, Faculty of Science, Al-Azhar University, to create the guava leaf extract. To get rid of any dirt or impurities, the leaves were meticulously cleaned with sterile Milli-Q water. Ten grams of these clean leaves were then cooked for approximately five minutes at 90 °C in 100 milliliters of distilled water. The mixture was allowed to cool before being filtered using Whatman No. 1 filter paper to create a transparent extract. The experiment’s subsequent procedures immediately made use of this freshly made leaf extract.

### Biosynthesis of silver oxide nanoparticles

The obtained guava leaf extract (0.1 g/mL) was gradually added to 100 mL of a 5 mM silver nitrate solution to create Ag₂O-NPs. At 60 °C, the reaction mixture was maintained. The solution turned brownish-yellow in about five minutes, which was an obvious indication that nanoparticles had formed and silver ions had been decreased. This change in color verified that Ag₂O-NPs were successfully biosynthesized^27^. After formation, the nanoparticles were kept in a dark environment until they were prepared for use in additional experiments.

#### Evaluation of silver oxide nanoparticle properties

The initial formation of Ag₂O-NPs was confirmed through a simple visual cue—a distinct color change in the reaction mixture^28^. To thoroughly characterize the nanoparticles, a combination of analytical techniques was employed.

 The synthesized nanoparticles underwent comprehensive characterization using various techniques: UV–Visible Spectrophotometry (Shimadzu) to assess optical properties, Fourier Transform Infrared Spectroscopy (FTIR) to identify functional groups and biomolecules involved in stabilization. Scanning electron microscopy (SEM), EDX and transmission electron microscopy (TEM) were used to evaluate the size and shape of the Ag₂O-NPs generated. Before imaging with magnifications ranging from ×50,000 to ×120,000 at an accelerating voltage of 80 kV, a drop of the nanoparticle dispersion was applied on carbon-coated copper grids and left to air dry. Gold-sputter-coated dried nanoparticle powder was positioned on aluminum stubs and analyzed at a 20 kV accelerating voltage at up to ×50,000 magnifications for SEM. In order to assess particle size distribution and colloidal stability, scale bars and magnification values were added to each TEM and SEM micrograph, as well as Dynamic Light Scattering (DLS) and Zeta Potential Analysis. Using ImageJ software, over 100 individual nanoparticles were measured for particle size analysis. The data were displayed as a histogram that displayed the size distribution.

### Test organisms

ATCC reference strains were obtained from American Type Culture Collection of *Salmonella typhimurium* ATCC 13311, *Escherichia coli* ATCC 25922, *Pseudomonas aeruginosa* ATCC 27853, and *Staphylococcus aureus* ATCC 29213 were used in assays to assess the antibacterial efficacy of the Ag₂O-NPs.

### Testing the antibacterial activity of silver oxide nanoparticles

#### Using the agar well diffusion method

Mueller-Hinton agar plates (90 mm in diameter) were inoculated with bacterial suspensions that were adjusted to the 0.5 McFarland standard (1.5 × 10^8^CFU/mL) in order to assess antibacterial activity. Under sterile circumstances, 100 µL of each suspension was equally distributed on the agar surface. 100 µL of Ag_2_O-NPs solution at the appropriate concentration was added to wells that were aseptically punched into the agar and had a diameter of 6 mm. After 30 min of diffusion at 4 °C, the plates were incubated for 18 to 24 h at 37 °C. Each run contained a growth control (inoculated agar without nanoparticles), a sterility control (uninoculated agar plate), and a negative/solvent control (sterile distilled water). Inhibition zones were measured in millimeters along two perpendicular axes and averaged for each assay, which was carried out in triplicate. The typical antibiotic comparator is 30 µg of chloramphenicol disks for each ATCC reference strain. For *Salmonella typhimurium* ATCC 13311, *Staphylococcus aureus* ATCC 29213, and *Escherichia coli* ATCC 25922, inhibition zones ≥ 18 mm were considered susceptible, 13–17 mm intermediate, and ≤ 12 mm resistant, according to CLSI breakpoints. Since there are no CLSI interpretation criteria for chloramphenicol, the observed zone widths for *Pseudomonas aeruginosa* ATCC 27853 were reported without category classification. To measure antibacterial activity, the diameter of each well and the surrounding zones where bacteria failed to grow (Inhibition zones) were recorded. Chloramphenicol (from Sigma-Aldrich) was used as positive control to compare how the bacteria responded to standard antibiotic. Every test was repeated three times to ensure accurate and reliable results^29^.

#### Minimum inhibitory concentration (MIC)

In accordance with known methods and CLSI guidelines, a broth microdilution method was employed to ascertain the degree to which Ag₂O-NPs and silver nitrate limit bacterial growth. Silver nitrate and gentamicin were used as positive controls. In this procedure, 96-well plates were used to gradually dilute the test compounds in Mueller-Hinton Broth. Each well was filled with a bacterial suspension that had been adjusted to meet a 0.5 McFarland standard, or roughly 10⁶ CFU/mL. The MIC was determined as the lowest concentration of the material that did not exhibit any discernible bacterial growth over a 24h incubation period at 37 °C^30^.

#### Minimum bactericidal concentration (MBC)

The minimum inhibitory concentration (MIC) of Ag₂O-NPs in sterile 96-well microplates was estimated using the broth microdilution method in compliance with CLSI guidelines. Mueller-Hinton broth was used to serially dilute the nanoparticles twice until the final well volume was 200 µL. To create a final inoculum of roughly 5 × 10^5^ CFU/mL per well, bacterial suspensions adjusted to 0.5 McFarland (≈ 1.5 × 10^8^CFU/mL) were added to each well and further diluted. MIC is the lowest dose at which no bacterial growth was detected following a 24h incubation period at 37 °C. 10 µL aliquots from the MIC well and two higher concentrations were streaked on Mueller-Hinton agar and incubated at 37 °C for 18 to 24 h in order to determine the minimum bactericidal concentration (MBC). The MBC was defined as the lowest concentration that resulted in ≥ 99.9% bacterial death. In every run, there were sterility control wells (broth only), solvent control wells (broth + solvent), growth control wells (bacteria + broth without nanoparticles), and positive controls (gentamicin and silver nitrate). All assays were carried out in triplicate^28^.

#### MTT cytotoxicity assay

The MTT test was used to evaluate the cytotoxicity of Ag₂O-NPs, which gauges cell viability by measuring mitochondrial metabolic activity. Using this method, the yellow, water-soluble tetrazolium salt (MTT) is enzymatically reduced to insoluble purple formazan crystals in metabolically active, viable cells. The number of living cells is directly correlated with the intensity of the purple hue as measured by spectrophotometry. Vero (normal) and HepG2 (cancerous) cell lines (bought from Science Way Co., Egypt) were incubated for 24 h at 37 °C with 5% CO₂ after 96-well plates were seeded with 1.5 × 10⁴ cells/mL in 100 µL of culture media. After preparation in 0.5% DMSO, Ag₂O-NPs were sterilized using 0.45 μm syringe filters and added in triplicate at doses ranging from 31.25 to 1000 µg/mL.

Following a 24 h exposure period, the growth media was replaced, and cells were cultivated for an additional four hours prior to the application of MTT. 100 µL of 1% HCl in isopropanol was used to dissolve the formazan crystals that had formed during the experiment. Absorbance at 570 nm was measured using a microplate reader. Cell viability was calculated using the formula (absorbance of treated sample / absorbance of control) × 100. The half-maximal inhibitory concentration (IC₅₀), or the concentration of Ag₂O-NPs required to reduce cell viability by 50%, was determined using the generated dose-response curve. To determine the IC₅₀ for Vero and HepG2 cell lines based on dose-response curves, nonlinear regression fitting (four-parameter logistic / sigmoidal dose-response) was performed in GraphPad Prism or a comparable application. All cytotoxicity experiments were performed in triplicate (*n* = 3 distinct trials), and the IC₅₀ values are shown as mean ± standard deviation (SD). The selectivity index (SI) was calculated as follows:

$$\:SI=\frac{IC50\left(Vero\right)}{IC50\left(HepG2\right)}$$

SI values were calculated from the mean IC₅₀ values and reported in the Results. Where relevant, statistical comparisons were performed and described in the figure/table legends^31^.

## Result

### Production and characterization of silver oxide nanoparticles

The reducing and stabilizing agents in this investigation were *Psidium guajava* leaf extract, which was used to successfully produce Ag₂O-NPs. At a 1000 µg/mL concentration, 100 mL of Ag_2_O-NPs suspension was created. The addition of the leaf extract caused a discernible color shift in the AgNO₃) solution from colorless to brown, visually confirming the synthesis. The creation of Ag₂O-NPs and the reduction of silver ions (Ag⁺) were visually shown by this color shift.

### UV–Visible absorption spectra

**Fig. 1:**
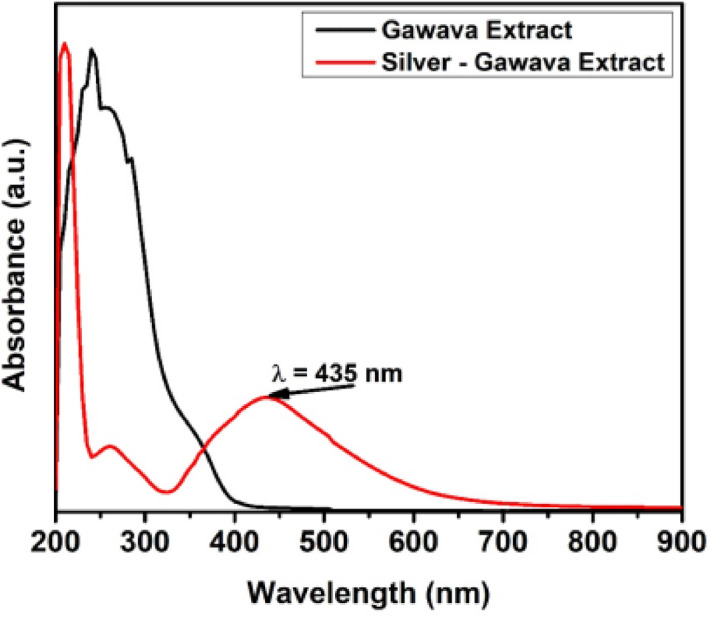
Surface plasmon resonance (SPR) absorption bands of Ag₂O-NPs capped with *Psidium guajava* leaf extract.

The biosynthesis of Ag₂O-NPs was carried out at 32 °C without light. Once more demonstrating the reduction of silver ions, a dark brown hue developed when the plant extract supernatant was added to the Ag⁺ solution. Surface plasmon resonance (SPR), a property of Ag_2_O-NPs, is activated, causing the solution to change from pale yellow to brown. The synthesis of Ag₂O-NPs was further confirmed using UV-Vis spectroscopy. A distinct SPR absorption peak at 435 nm verified the existence of colloidal Ag_2_O-NPs (Fig. 1).

### Dynamic light scattering (DLS) and zeta potential analysis

The surface characteristics and colloidal stability of the produced Ag_2_O-NPs were evaluated over time using zeta potential measurements and dynamic light scattering (DLS) (Table 1). The Ag₂O-NPs showed a polydispersity index (PDI) of 0.368 and an average hydrodynamic diameter (HD) of roughly 57.25 ± 24.25 nm after *Psidium guajava* leaf extract was added to the AgNO₃ solution. The produced nanoparticles’ zeta potential was found to be -0.27 mV, which indicates negligible electrostatic stabilization. This low value suggests that the nanoparticles are not electrostatically stable; instead, their apparent short-term dispersion can be attributed to steric stabilization by phytochemicals derived from *Psidium guajava* extract, consistent with their classification as Ag₂O-NPs, as seen in Table 1.

**Table 1:** Colloidal and surface properties of as-prepared Ag₂O-NPs.

Sample	Colloidal Properties Dynamic Light Scattering (DLS)		Zeta Potential, mV
	H_D_, nm	PDI	
Ag_2_O- NPs	57.25 ± 24.25	0.368	– 0.275 ± 4.26

### FTIR spectroscopic analysis

**Fig. 2:**
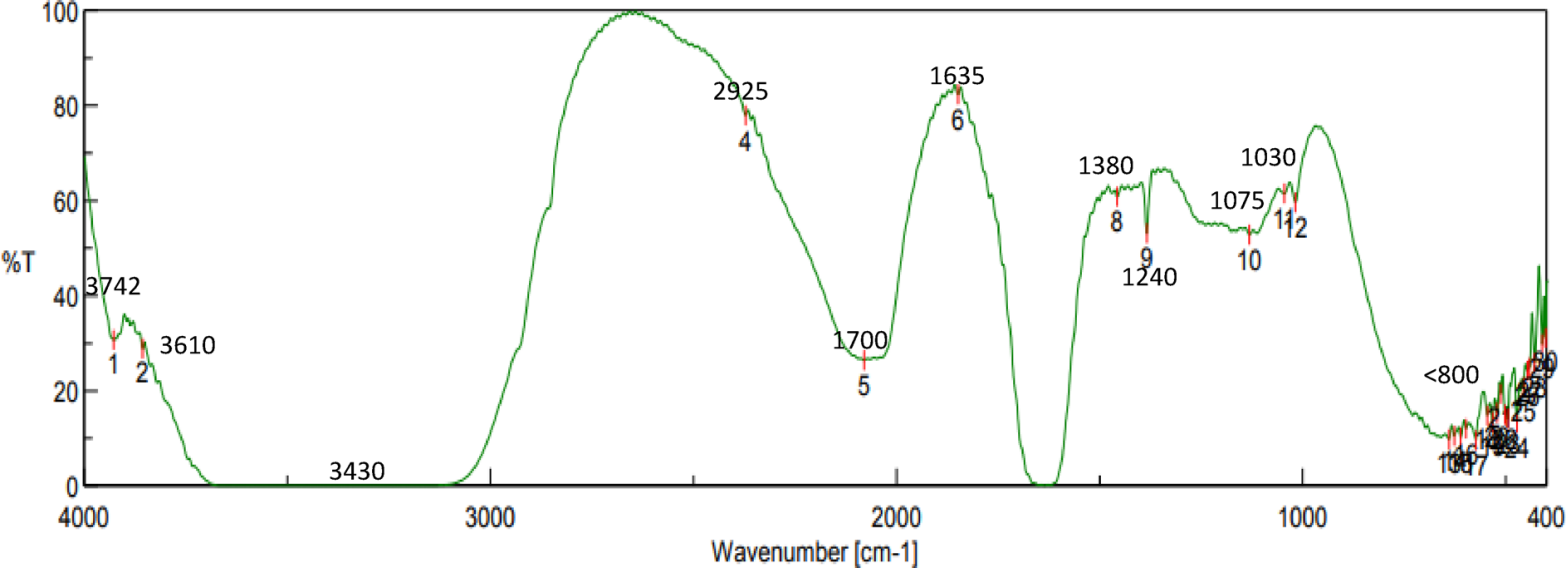
FTIR spectra of *Psidium guajava* leaf extract and the synthesized Ag₂O-NPs.

Using *P. guajava* leaf extract, Fourier Transform Infrared (FTIR) spectroscopy was utilized to determine the functional groups involved in the production and stabilization of Ag₂O-NPs (Fig. 2). The broad band at roughly 3300 cm⁻¹ is linked to O–H/N–H stretching of phenols, flavonoids, or proteins, whereas the peak at roughly 1700 cm⁻¹ is attributed to C=O stretching vibrations of aldehydes, ketones, or carboxylic acids. The C–O and C–N stretching in alcohols, esters, or polysaccharides is associated with bands in the 1200–1000 cm⁻¹ region, while aromatic C= C or amide I vibrations are linked to the 1600 cm⁻¹ band. Importantly, bands that show how silver oxide interacts with biomolecules are found below 800 cm⁻¹ and related to metal–oxygen vibrations. These results lend credence to the hypothesis that the phytochemicals in the extract function as capping and reducing agents to mediate the synthesis and stabilization of Ag₂O-NPs as shown in (Fig. 2).

### XRD analysis

**Fig. 3:**
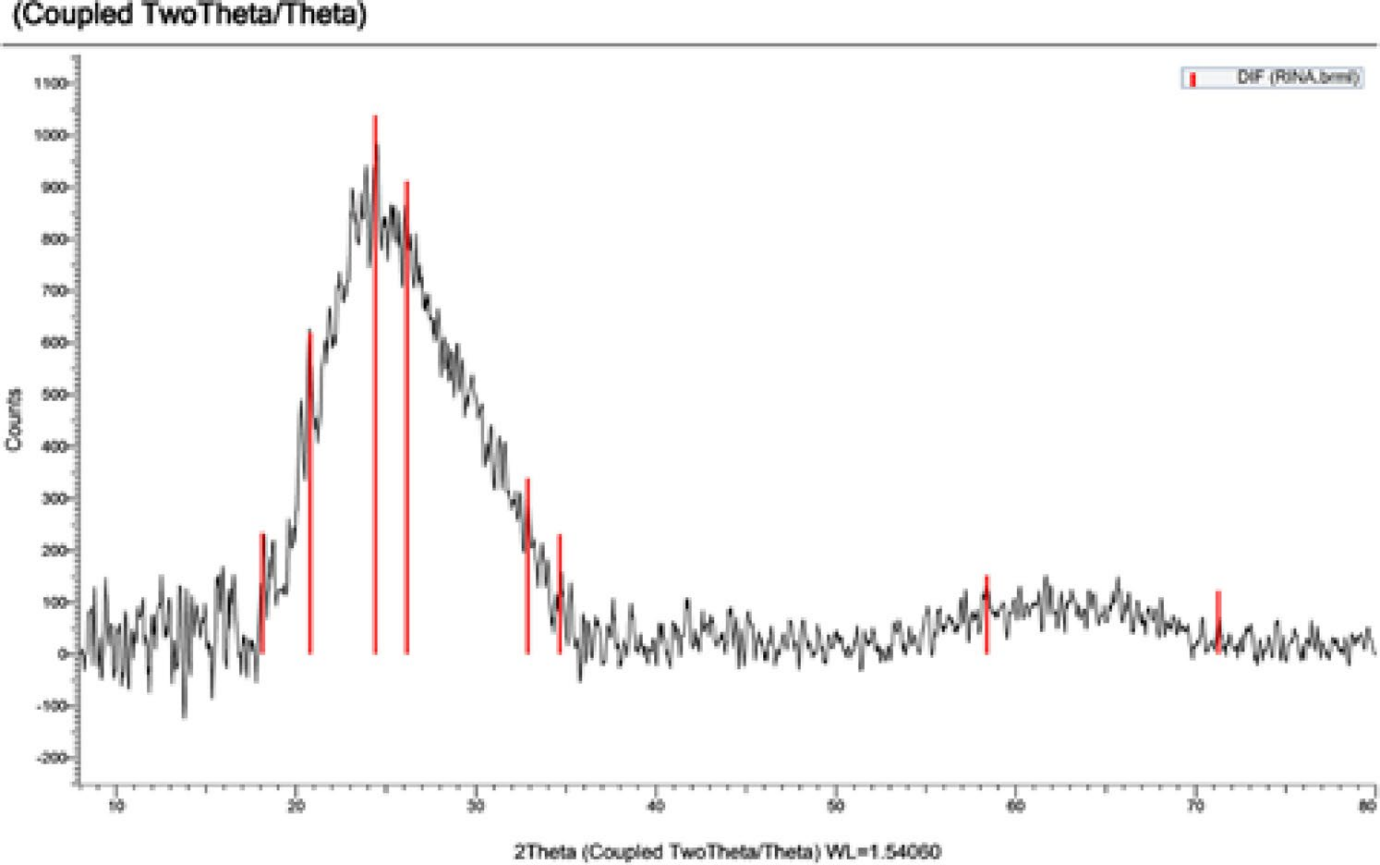
XRD pattern of Ag₂O-NPs synthesized using *P. guajava* leaf extract.

Figure 3 displays the XRD pattern of nanoparticles made with *Psidium guajava* leaf extract; the peak list and matching raw diffractogram are included in Supplementary Table S1. At 2θ values of 18.1°, 20.8°, 24.4°, 26.1°, 32.9°, 34.6°, 58.3°, and 71.2°, the diffraction peaks were detected. The creation of crystalline Ag_2_O-NPs is confirmed by these peaks, which are consistent with the standard diffraction data for silver oxide (AgO, JCPDS card no. 41-11104). The crystalline structure of the oxide phase and potential organic residues from the plant extract functioning as capping agents may be the causes of the many peaks in the lower angle area (18.1–26.1°).

### SEM and EDX analysis

**Fig. 4:**
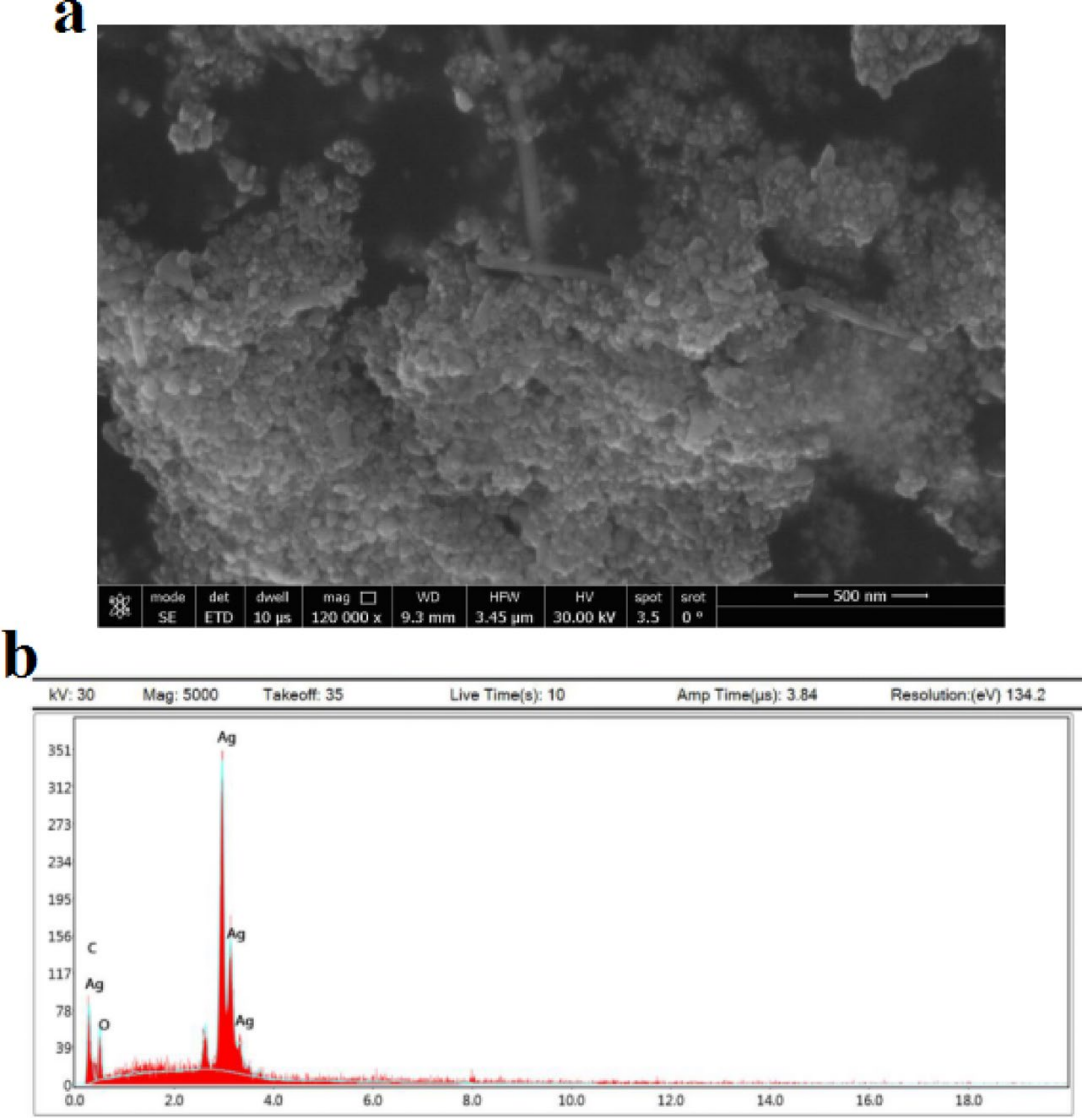
a: SEM image and b: EDX of Ag₂O-NPs produced by *Psidii guajavae folium* extract,

Scanning electron microscopy (SEM) image (Fig. 4a) showed that the nanoparticles were mostly spherical in shape. The crystalline nature of the Ag_2_O-NPs was confirmed by powder X-ray diffraction (XRD) analysis, which also determined the average crystallite size to be around 27 nm. EDX analysis confirmed the elemental composition of the biosynthesized Ag₂O-NPs. Silver was identified as the predominant element, accounting for 58.4% of the total weight, followed by oxygen (35.73%) and a minor contribution from carbon (5.84%). These findings indicate that silver oxide is the major component of the nanoparticles, while the presence of oxygen further supports the formation of Ag₂O-NPs, and the trace carbon content may be attributed to phytochemical residues from the *P.guajava* leaf extract (Fig. 4b), which clearly indicates that organic molecules from *P.guajava* leaf extract were crucial in stabilizing and lowering the Ag₂O-NPs.

### Transmission electron microscopy (TEM)

**Fig. 5:**
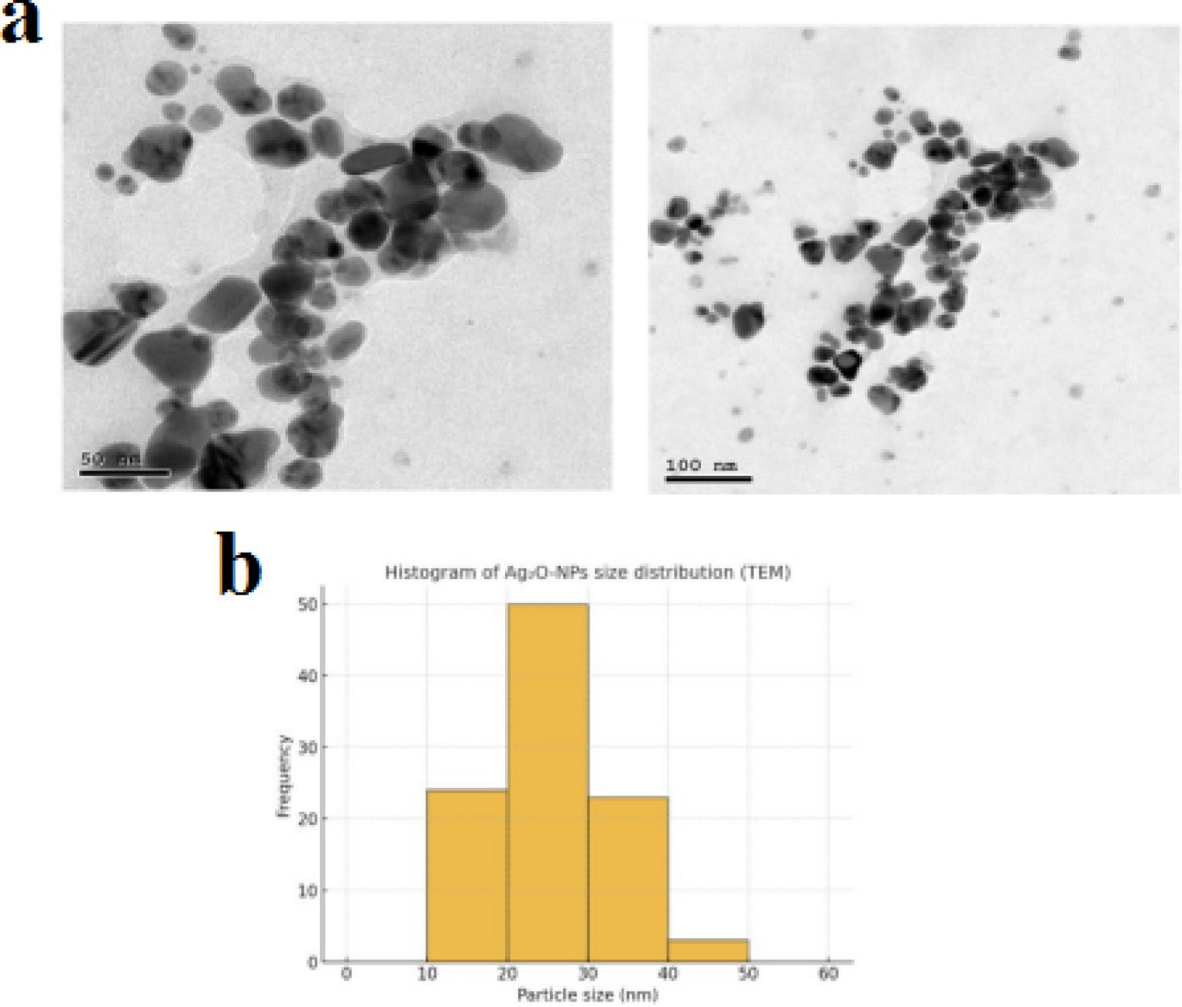
(**a**) TEM images of green-synthesized Ag₂O-NPs using *P. guajava* leaf extract, showing spherical morphology, (**b**) TEM micrograph and corresponding histogram showing the particle size distribution of Ag₂O-NPs. The nanoparticles exhibited sizes ranging between 10 and 45 nm, with an average of ~ 25 ± 5 nm.

The biosynthesized Ag_2_O-NPs were primarily spherical in shape and widely distributed, according to Transmission Electron Microscopy (TEM) pictures. Over 100 nanoparticles were tested in order to further examine their size distribution, and the results were presented as a histogram (Fig. 5a, b). The average particle size was approximately 25 ± 5 nm, with the majority of the particles lying within the 20–30 nm range. The particle sizes ranged from 10 to 45 nm. This limited dispersion suggests that the produced nanoparticles are comparatively homogeneous (monodispersed) as shown in Supplementary Figure S1.

### Antibacterial activity

**Fig. 6:**
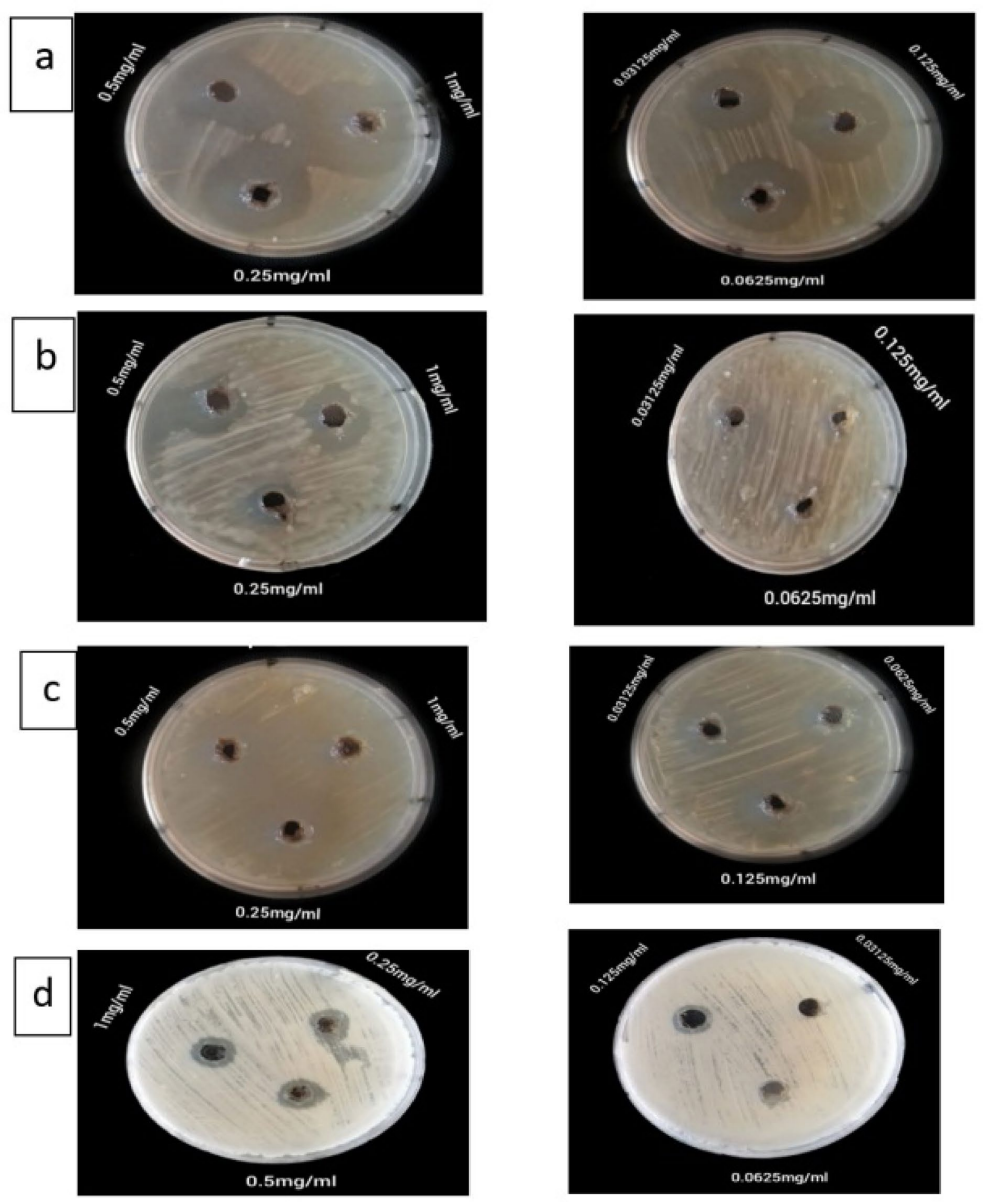
Antibacterial activity of Ag₂O-NPs against four selected clinical strains by agar well diffusion method (**a**) *Pseudomonas aeruginosa*, (**b**) *Salmonella typhimurium*, (**c**) *Staphylococcus aureus*, (**d**) *E. coli*.

*Pseudomonas aeruginosa*, *Staphylococcus aureus*, *Salmonella typhimurium*, and *Escherichia coli* were the four clinical isolates against which the Ag₂O-NPs shown strong, dose-dependent antibacterial activity. With a broad inhibitory zone of 32 mm at 1 mg/mL and a zone of 19 mm at the lowest dosage tested (31 µg/mL), *P. aeruginosa* was the most susceptible of these. With inhibitory zones that ranged from 15 mm at the lowest concentration to 31 mm at 1000 µg/mL, *S. aureus* also shown remarkable sensitivity. Only at higher concentrations (25 mm at 1000 µg/mL) did *S. typhimurium* exhibit action; at lower concentrations (less than 250 µg/mL), no discernible inhibition was seen. The least responsive was *E. coli*, which showed no activity at 31 µg/mL and a maximal inhibition zone of 14 mm (Table 2; Fig. 6).

**Table 2:** Silver oxide nanoparticles’ ability to inhibit the growth of selected reference strains.

Concentration of Silver oxide nanoparticles in µg/mL:	Antibacterial activity of silver oxide nanoparticles against selected reference strains in mm			
	*P*. *aeruginosa* ATCC 27853	*S. typhimurium* ATCC 13311	*S. aureus* ATCC 29213	*E. coli* ATCC 25922
1000 µg/mL	32 ± 1.212	25 ± 1.050	31 ± 0.656	14 ± 0.458
500 µg/mL	28 ± 0.700	19 ± 0.173	29 ± 0.557	12 ± 0.819
250 µg/mL	26 ± 0.794	11 ± 0.361	27 ± 0.513	12 ± 0.914
125 µg/mL	24 ± 0.265	0.0	20 ± 0.964	11 ± 0551
62.5 µg/mL	21 ± 0.416	0.0	18 ± 0.436	9 ± 0.351
31 µg/mL	19 ± 0.306	0.0	15 ± 0.917	0.0
Chloramphenicol (30 µg)	— (no CLSI breakpoint)	≥ 18 (S); 13–17 (I); ≤12 (R)	≥ 18 (S); 13–17 (I); ≤12 (R)	≥ 18 (S); 13–17 (I); ≤12 (R)

µg = microgram, mL = milliliter, mm = millimeter, R = Resistance, I = Intermediate, S = Sensitive.

### MIC, MBC, and MIC/MBC ratio

For every studied bacterial strain, the Ag₂O-NPs Minimum Inhibitory Concentration (MIC) varied from 250 to 31.2 µg/mL. The result shows that the Minimum Bactericidal Concentration (MBC) values had a bactericidal effect, and the MIC/MBC ratios supported cidal action, as demonstrated in Table 3.

**Table 3:** Minimum inhibitory concentration (MIC), minimum bactericidal concentration (MBC), and MBC/MIC ratio of Ag₂O-NPs (µg/mL) against four clinical strains.

Clinical strains	MBC	MIC	MBC/ MIC ratio
*Pseudomonas aeruginosa*	250	125	2 (+)
*Salmonella typhimurium*	62.5	31.2	2(+)
*E.coli*	500	250	2(+)
*Staphylococcus aureus*	500	250	2(+)

### Cytotoxicity in normal cells (Vero cell Line)

**Fig. 7:**
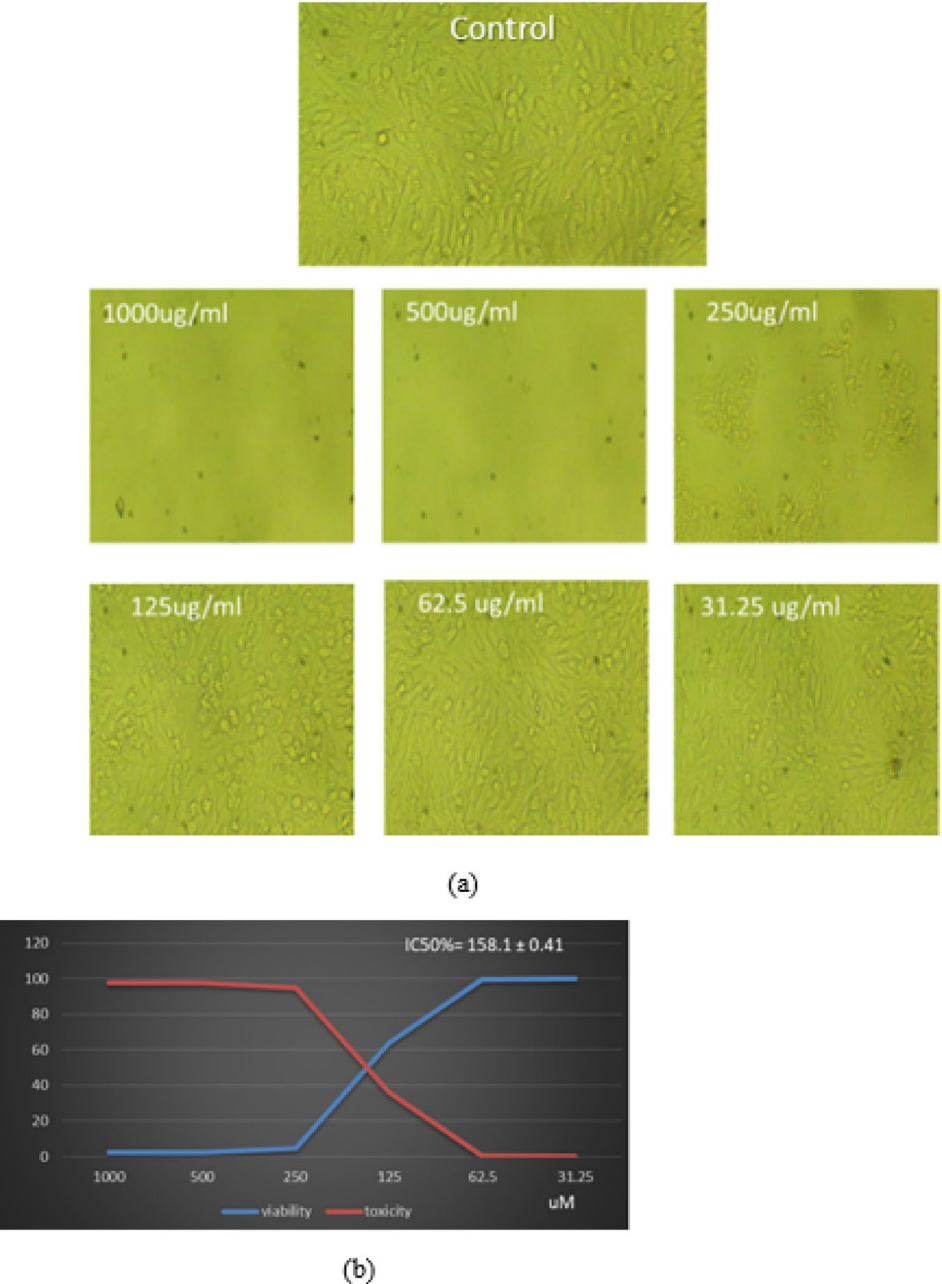
Cytotoxicity evaluation of Ag₂O-NPs on Vero cells using MTT assay. (**a**) Microscopic images of Vero cells showing untreated control and treated cells at different nanoparticle concentrations. (**b**) Dose–response curve representing cell viability (%) and IC₅₀ value calculated for Ag₂O-NPs.

The cytotoxic impact of Ag₂O-NPs on Vero cells was assessed over a range of concentrations (31.25 to 1000 µg/mL). As shown in (Fig. 7a), apoptotic morphological changes including cell shrinkage and rounding were observed in Vero cells treated with Ag₂O-NPs. Cell viability decreased in a dose-dependent manner: from 99.95% at the lowest dose to 63.97%, then drastically dropping to 4.90%, 2.47%, and 2.43% at higher concentrations. The IC₅₀ value the consent ration at which cell viability is reduced by 50% was determined to be 158.1 ± 0.41 µg/mL ( Fig. 7b).

### Cytotoxicity in HepG2 cancer cells

**Fig. 8:**
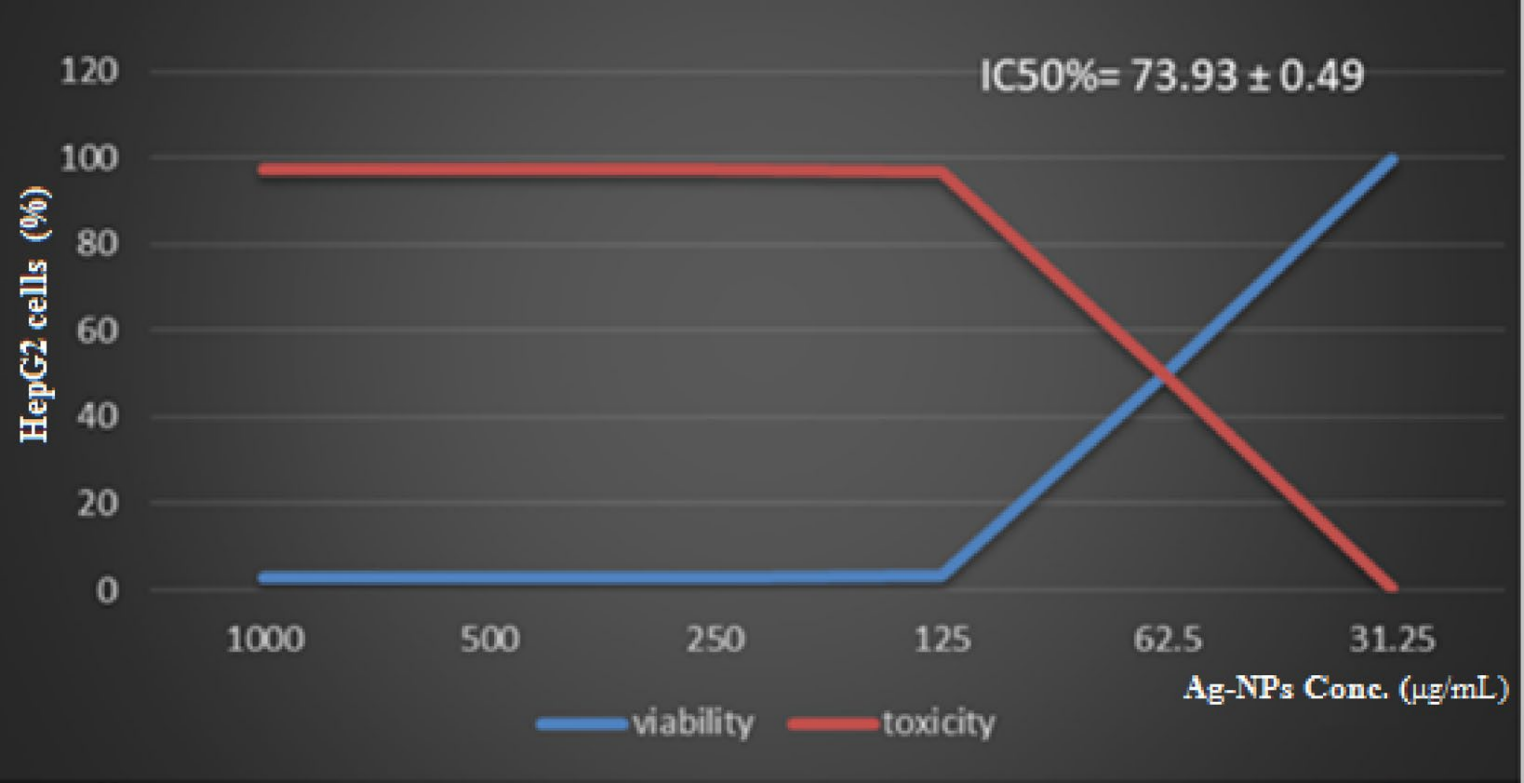
Dose–response curve representing cell viability (%) and IC₅₀ value calculated for Ag₂O-NPs.

Silver oxide nanoparticles were also tested against HepG2 liver cancer cells over the same concentration range. A steep decline in cell viability was observed: 99.48%, 50.45%, 3.22%, 2.93%, 2.64%, and 2.64% as concentrations increased (Table 4) (Fig. 8). The IC₅₀ value for HepG2 cells was 73.93 ± 0.49 µg/mL, suggesting a stronger cytotoxic effect on cancer cells compared to normal cells. The IC₅₀(Vero)/IC₅₀(HepG2) selectivity index (SI) was 2.14, indicating roughly twofold greater toxicity toward cancer cells than normal cells.

**Table 4:** Cellular interaction of Ag₂O-NPs synthesized using *p. guajava* extract with HepG2 cancer cells.

HepG2 cell		
Ag₂O-NPs Conc. ug mL^− 1^	Viability (%)	Toxicity (%)
Control	100	0
31.25	99.48	0.52
62.2	50.45	49.55
125	3.22	96.78
250	2.93	97.07
500	2.64	97.35
1000	2.64	97.35

IC_50_ = 73.93 ± 0.49 ug mL^− 1^

## Discussion

UV-Vis, FTIR, XRD, SEM, and TEM investigations verified the successful green synthesis of Ag₂O-NPs utilizing *P. guajava* leaf extract. Characteristic FTIR peaks and the surface plasmon resonance band at 435 nm provided evidence for the role of phytochemicals in stability and reduction. In line with previous studies, TEM and SEM micrographs revealed primarily spherical particles of 25–30 nm, whereas XRD and SAED patterns validated the crystalline oxide phase^32,33^. These results are consistent with earlier research showing that extracts rich in phytochemicals serve as capping and reducing agents during the production of nanoparticles^32,34^.

According to Fayyadh and Alzubaidy^35^, leftover oxygen, water, and reactive phytochemicals can help AgO layers grow on metallic cores in green synthesis systems, which can alter diffraction signatures and crystalline quality. Similarly, Mani et al.^36^, notes that the resultant nanoparticles may mostly display oxide structures rather than pure metal when stronger reducing agents are not present or under mild circumstances. The contribution of organic molecules from the extract to stabilizing the surface of the nanoparticle is further supported by the presence of carbon and oxygen in the EDX spectrum.

The produced Ag₂O-NPs showed broad-spectrum antibacterial action, with *S. aureus* and *P. aeruginosa* showing the highest effects. Their potential as antibacterial agents are supported by the MIC values (31.2–250 µg/mL), which are within the range documented in related research employing plant-mediated Ag₂O-NPs. Variations in cell wall construction, permeability, and efflux mechanisms can all be linked to differences in bacterial susceptibility. In line with previous research on green-synthesised silver oxide nanoparticles, the measured MIC/MBC ratios suggested bactericidal activity. Against several clinical strains, the produced Ag₂O-NPs showed a potent dose-dependent antibacterial activity. The strong antibacterial qualities of the nanoparticles were highlighted by the increase in inhibition zones with their concentration. These results are consistent with Velsankar et al.‘s research^37^, who reported inhibition zones of 28 ± 1.1 and 26 ± 0.8 mm against *S. aureus* and *S. typhi* using Ag₂O-NPs synthesized with grain of *Panicum miliaceum* extract. Patel and Joshi^38^ observed high inhibition zone at 16.9 mm against *S. aureus* using Ag₂O-NPs synthesized with *Salix integra plant*.

Ag₂O-NPs had a greater effect on HepG2 liver cancer cells (IC₅₀ = 73.93 µg/mL) than on normal Vero cells (IC₅₀ = 158.1 µg/mL), according to cytotoxicity study. A minor safety margin is indicated by the computed selectivity index (SI = 2.14), which suggests preferential toxicity toward cancer cells but also emphasizes the need for additional confirmation. Though they are still theories until they are verified experimentally, the anticancer activity may entail processes suggested in earlier research, such as ROS production, mitochondrial malfunction, and DNA damage resulting in apoptosis^39–41^.

A moderate level of selectivity is indicated by a SI value of 2.14. The safety margin is narrow, and more research on other normal cell lines and in vivo models is required to confirm therapeutic promise, even though this suggests some preferential toxicity toward cancer cells^42–44^.

While the present findings highlight the antimicrobial and anticancer potential of biosynthesized Ag₂O-NPs, further studies are required to confirm their clinical applicability. In vivo evaluations should be performed to assess pharmacokinetics, biodistribution, and long-term safety. Additional cytotoxicity assays on multiple normal human cell lines will be essential to better define the therapeutic window. Mechanistic studies focusing on ROS generation, apoptosis, and DNA damage are also needed to clarify molecular pathways. Moreover, nanoparticle formulation into biocompatible delivery systems could enhance stability, selectivity, and targeted action, paving the way for potential translational applications in infectious disease management and cancer therapy.

## Conclusion

This work highlights the successful green synthesis of silver oxide nanoparticles (Ag_2_O-NPs) employing leaf extract from *Psidium guajava* as a natural stabilizing and reducing agent. The resulting nanoparticles were spherical, crystalline, and nanoscale in size. Additionally, they showed strong antibacterial action against *Pseudomonas aeruginosa* and *Staphylococcus aureus*. Additionally, they showed selective cytotoxicity, harming HepG2 liver cancer cells more than they did healthy Vero cells. There are still certain limitations, though. This study only tested a single cancer cell line and a small number of bacterial strains; further investigation is required to confirm the nanoparticles’ long-term physicochemical stability. Future studies should be done in vivo toxicity and efficacy tests, incorporate a variety of cancer models, and broaden the range of bacterial pathogens examined. Possible clinical translation will also be supported by clarifying the molecular mechanisms behind antibacterial and anticancer action. Crucially, the synthesis method is inexpensive, environmentally benign, and devoid of dangerous chemicals, which makes it sustainable and advantageous to society. These characteristics lessen environmental hazards and aid in the advancement of reasonably priced nanomedicine. This green synthesis method has a lot of potential for scale-up and commercialization due to its ease of use, repeatability, and economic feasibility, opening the door for its use in cancer treatment and antimicrobial therapy.

## Supplementary Information

Below is the link to the electronic supplementary material.

Supplementary Material 1

## Data Availability

Additional datasets and figures supporting the findings of this study are provided as Supplementary Materials. These include extended data related to antibacterial activity and cytotoxicity assays.
