# Supplementary material for: Evaluation of antibacterial and cytotoxic effects of silver oxide nanoparticles synthesized from Psidium Guajava
Source: Sci Rep. 2025 Nov 25;15:41763. doi: 10.1038/s41598-025-24924-6 (PMC12647600; doi:10.1038/s41598-025-24924-6)
Supplement: Supplementary file 1 — Supplementary Material 1 [file 41598_2025_24924_MOESM1_ESM.docx]

**Table S1:** XRD peak list and tentative assignments for Ag₂O-NPs.

| **2θ (°)** | **d-spacing (Å)** | **Relative intensity (%)** | **Phase assignment (JCPDS)** | **Remark** |
| --- | --- | --- | --- | --- |
| 18.1 | 4.89 | 22.5 | Organic residue / Ag₂O | Low-angle peak, likely plant capping or oxide |
| 20.8 | 4.27 | 59.6 | Ag₂O (JCPDS 41-1104) | Strong oxide reflection |
| 24.4 | 3.64 | 100.0 | Ag₂O (JCPDS 41-1104) | Dominant oxide peak |
| 26.1 | 3.40 | 87.8 | Ag₂O (JCPDS 41-1104) | Matches oxide phase |
| 32.9 | 2.72 | 32.7 | Ag₂O (JCPDS 41-1104) | Oxide reflection |
| 34.6 | 2.59 | 22.3 | Ag₂O (JCPDS 41-1104) | Oxide reflection |
| 58.3 | 1.58 | 14.6 | Possible Ag / Ag₂O | Weak, may overlap with metallic Ag |
| 71.2 | 1.32 | 11.7 | Ag (311) | Weak reflection of metallic Ag |


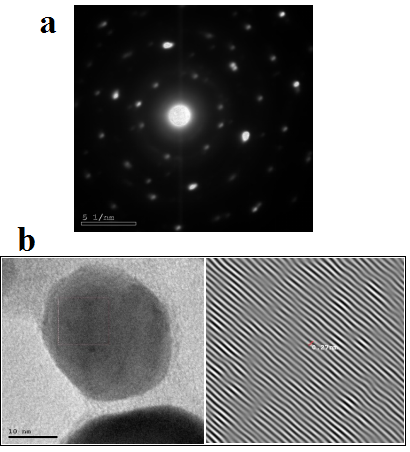


**Figure S1:** a: SAED image and b: TEM micrographs with high resolution for as-prepared Ag₂O-NPs.

Control
